# Necrotizing fasciitis caused by a primary appendicocutaneous fistula

**DOI:** 10.1007/s00595-012-0140-x

**Published:** 2012-02-10

**Authors:** Makoto Takeda, Yukihiro Higashi, Tuyoshi Shoji, Takanori Hiraide, Hirotoshi Maruo

**Affiliations:** Department of Surgery, Shizuoka City Shimizu Hospital, 1231 Miyakami, Shimizu-Ku, Shizuoka 424-8636 Japan

**Keywords:** Necrotizing fasciitis, Appendicocutaneous fistula, Appendicitis

## Abstract

We report a case of necrotizing fasciitis in the loin of a 76-year old man with several coexisting or past health issues, including diabetes mellitus, hypertension, alcohol-related liver cirrhosis, gastrectomy for gastric cancer, subarachnoid hemorrhage, normal pressure hydrocephalus, and cerebral infarction. Incision of the necrotizing fasciitis was successful, but it revealed an appendicocutaneous fistula; thus, we performed appendectomy and fistulectomy. We think that the necrotizing fasciitis was caused by appendicitis perforation involving the retroperitoneum, inducing the formation of an appendicocutaneous fistula. Necrotizing fasciitis and appendicocutaneous fistulae are rare complications of appendicitis. Moreover, to our knowledge, this is the first report of fluoroscopic examination demonstrating that a primary appendicocutaneous fistula had caused necrotizing fasciitis. Our search of the literature found 12 cases of necrotizing fasciitis caused by preoperative appendicitis. We discuss the characteristics and findings of these cases.

## Introduction

Necrotizing fasciitis is a potentially fatal soft tissue infection associated with high mortality (6–76%) [1]; the necessity of early aggressive surgical debridement for necrotizing fasciitis is well documented. When a pathogen enters the subcutaneous space, necrotizing fasciitis can occur. Although appendicitis is one of the soft tissue injuries included in its etiology, appendicitis resulting in necrotizing soft tissue infection is extremely rare and often fatal [2]. We report a case of a primary appendicocutaneous fistula causing necrotizing fasciitis, which is an exceptionally rare event [3].

## Case report

**Fig. 1:**
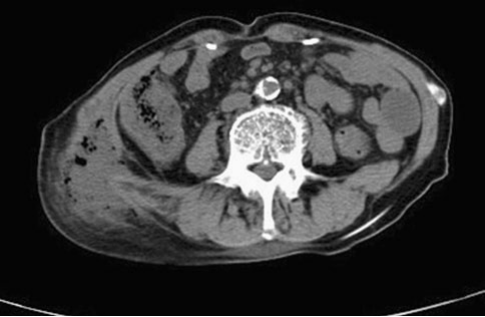
Abdominal computed tomography scan (*horizontal image*) showed gas and fluid collection from the subcutaneous layer to the retroperitoneal cavity, with inflammation extending into the adjacent soft tissue near the loin

**Fig. 2:**
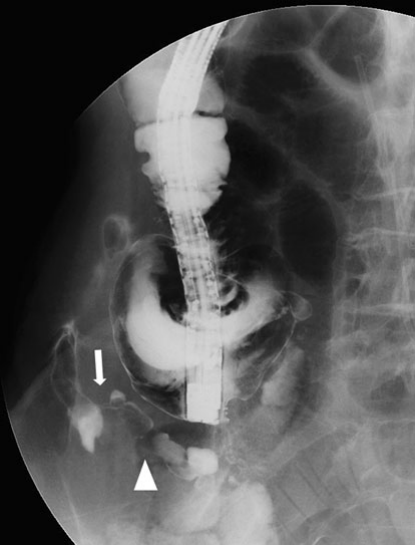
Fluoroscopic-aided colonoscopy showed the fistula (*arrow*) enhanced between the appendix (*arrowhead*) and the incised tissue

The patient was a 76-year-old man whose chief complaint was difficulty in walking and pain in his right loin. His coexisting disorders included diabetes mellitus, hypertension, and alcoholic liver cirrhosis. He also had a history of gastrectomy for gastric cancer, subarachnoid hemorrhage, normal pressure hydrocephalus, and cerebral infarction. On admission, his body temperature was 35.9°C; blood pressure, 113/80 mmHg; and pulse rate, 95 beats/min. He reported tenderness in his right loin, but there were no reddish areas or swelling. His laboratory data on admission were as follows: white blood cell count, 460 × 10^2^/μl (neutrophils: 98.5%); hemoglobin, 7.5 g/dl; creatine, 3.5 mg/dl and C-reactive protein, 25.7 mg/dl. Abdominal computed tomography (CT) showed gas and fluid collection extending from the subcutaneous layer of his right loin to his retroperitoneal cavity (Fig. 1). Based on these findings, we diagnosed necrotizing fasciitis. No connection was found between the necrotizing fasciitis and the gut and his appendix was not swollen. Therefore, a 10-cm incision was made in his right loin. Pus and foul-smelling gas were released, and the subcutaneous fat was debrided. Bacterial culture of the pus grew *Peptostreptococcus micros*, *Peptostreptococcus* sp., *Prevotella disiens*, and *Escherichia coli*. Antibiotic sensitivity tests for meropenem hydrate and clindamycin hydrochloride were positive. We irrigated the incised tissue and gave systematic antibiotic therapy (meropenem hydrate: 1 g/day, clindamycin hydrochloride: 1.2 g/day) for 10 days. His inflammatory response improved; however, the underlying cause of his condition could not be investigated effectively because he suffered epileptic seizures. About 3 months after his admission, sordes were discharged from the affected body part. Subsequently, an appendicocutaneous fistula was revealed by fluoroscopy-aided colonoscopy (Fig. 2). An open laparotomy revealed strong adhesions between the terminal ileum and retroperitoneum. We found that the distal end of the vermiform appendix had adhered to the retroperitoneum and a fistula had formed. Thus, we performed appendectomy and fistulectomy. The pathohistological diagnosis was chronic acute phlegmonous appendicitis.

His postoperative course was uneventful; however, on postoperative day 35, a urinary tract infection developed and methicillin-resistant *staphylococcus aureus* sepsis ensued, resulting in the death of multiple organ failure on postoperative day 48.

## Discussion

Necrotizing fasciitis is a rare soft tissue infection, which involves the superficial fascia, subcutaneous fat, and deep fascia. Giuliano et al. divided necrotizing fasciitis into two distinct groups, based on his analysis of bacteriologic culture results obtained from affected patients: Type 1, which is polymicrobial and involves non-group A streptococci plus anaerobes and/or facultative anaerobes and also often involves enterobacteriaceae; Type 2, also known as hemolytic streptococcal gangrene, in which the pathogen population is composed of group A β-hemolytic streptococci alone or in combination with a *staphylococcus* bacterium [2]. Necrotizing fasciitis can occur in any region of the body, but is found most commonly in the abdominal wall, extremities, and perineum. Involvement of the abdominal wall is usually a postoperative complication of abdominal surgery. Its reported causes include appendicitis, colocutaneous fistula, incarcerated hernia, perforated viscus, renal calculi after abdominal injury, and postoperative complications [1, 2, 4]. The case in this report was classified as type 1 and involved necrotizing fasciitis as a preoperative complication of appendicitis. We found only 12 case reports of patients with necrotizing fasciitis caused by appendicitis published in the English literature and cited in Pub Med (Table 1) [3, 5–15]. Four of these patients were male and nine, female, and their ages ranged from 28 to 85-years old (mean age: 66-years old). Seven of the 12 patients had a significant medical history (53.8%). It is evident that the disease occurs more frequently in patients with complications, but it can even in young, healthy individuals. Four (30.8%) of the patients died, reinforcing that the condition is associated with high mortality.

**Table 1:** Cases of necrotizing fasciitis caused by preoperative appendicitis reported in the English literature

No.	Author	Year	Sex	Age	Medical history/coexisting disease	Appendix condition	Results
1	Mazza [5]	1987	Female	59	None	Perforated appendicitis	Death
2	Guirguis [6]	1989	Female	80	Atrial fibrillation, hypertension, non-insulin-dependent diabetes, prescribed four drugs	Gangrenous appendix in an incarcerated femoral hernia	Alive
3	Jacobs [7]	1993	Female	44	Not described	Perforated appendicitis	Alive
4	Gerber [8]	1994	Male	85	Alzheimer’s dementia	Perforated appendicitis	Alive
5	Bobrow [9]	1996	Male	63	Hypertension, congestive heart failure, depression, insulin-requiring diabetes mellitus	Perforated appendicitis with abscess formation	Death
6	Groth [10]	1999	Female	49	Hypertension	Perforated appendicitis	Alive
7	Harwant [11]	2001	Female	66	Not described	Phlegmonous appendicitis	Death
8	Awe [3]	2003	Female	28	Not described	Perforated appendicitis with an appendicocutaneous fistula	Alive
9	Mukoyama [12]	2003	Male	77	Depression	Necrotizing appendicitis	Alive
10	Marron [13]	2005	Female	67	Not described	Incarceration of an inguinal hernia containing the appendix alone (Amyand’s hernia)	Alive
11	Penninga [14]	2006	Female	33	None	Perforated appendicitis	Alive
12	Chen [15]	2010	Female	76	Congestive heart failure, chronic obstructive pulmonary disease, chronic renal insufficiency	Perforated appendicitis with abscess formation	Alive
13	Our case	2010	Male	76	Diabetes mellitus, alcoholic liver cirrhosis, hypertension, cerebral infarction, subarachnoid hemorrhage, normal pressure hydrocephalus, gastric cancer	Perforated appendicitis with an appendicocutaneous fistula	Death

A diagnosis of necrotizing fasciitis is primarily based on the clinical and physical examinations. Patients complain of severe pain, and the characteristic examination features include edema and tenderness extending beyond the limits of cutaneous erythema, crepitus, and skin vesicles. Our patient did not complain of the typical symptoms so diagnosis was difficult using only clinical and physical examinations; however, the CT examination is very useful for confirming the diagnosis of necrotizing fasciitis.

Kjellman defined an appendiceal fistula as the primary perforation of the appendix into an adjacent hollow viscus or the skin and excluded fistulae resulting as sequelae of surgically treated appendicitis [16]. The bladder is the organ most commonly associated with appendix fistulae. Appendicocutaneous fistulae are a rare form of enterocutaneous fistulae, and very few cases have been reported [17, 18]. We did not investigate the cause of our patient’s condition, but concentrated on treating his necrotizing fasciitis and many complications. Based on the bacterial culture results and the presence of a fistula, we assume that acute appendicitis perforated the retroperitoneum and formed an appendicocutaneous fistula, resulting in necrotizing fasciitis. Considering the high rate of complications shown in Table 1, we speculate that our patient’s state of ill health predisposed to the acute appendicitis causing the fistula and necrotizing fasciitis. Prompt incision and drainage of the collected fluid and antibiotics is considered the best treatment strategy. The cause of necrotizing fasciitis must be investigated after early debridement of all necrotic tissue and drainage of pus.

In conclusion, appendicocutaneous fistula induced by appendicitis perforating the retroperitoneum is a rare cause of necrotizing fasciitis. Surgical debridement should be performed for the necrotizing fasciitis, the cause of which must be investigated after the patient’s condition has improved. Fluoroscopic-aided colonoscopy is very useful for investigating the cause of necrotizing fasciitis. Acute appendicitis is one of the most common diseases encountered in clinical practice; however, we should bear in mind that unusual complications can occur, such as necrotizing fasciitis and gangrenous intrathoracic appendicitis caused by incarceration of diaphragmatic hernia, as reported by Schellhaas et al. [19].
